# Folic Acid and PEI Modified Mesoporous Silica for Targeted Delivery of Curcumin

**DOI:** 10.3390/pharmaceutics11090430

**Published:** 2019-08-23

**Authors:** Xiaoxiao Sun, Nan Wang, Li-Ye Yang, Xiao-Kun Ouyang, Fangfang Huang

**Affiliations:** School of Food and Pharmacy, Zhejiang Ocean University, Zhoushan 316022, China

**Keywords:** curcumin, mesoporous silica nanoparticles, functional, delivery, cancer

## Abstract

Nano anti-cancer drug carriers loaded with antineoplastic drugs can achieve targeted drug delivery, which enriches drugs at tumor sites and reduces the toxic side effects in normal tissues. Mesoporous silica nanoparticles (MSN) are good nano drug carriers, as they have large specific surface areas, adjustable pore sizes, easily modifiable surfaces, and good biocompatibility. In this work, polyethyleneimine (PEI) grafted MSN were modified with folic acid (FA) as an active target molecule using chemical methods. The product was characterized by SEM, TEM, Zetasizer nano, FTIR, and an N_2_ adsorption and desorption test. MSN-PEI-FA are porous nano particles with an average particle size of approximately 100 nm. In addition, the loading rate and release behavior of MSN-PEI-FA were studied with curcumin as a model drug. The results show that when loading curcumin to MSN-PEI-FA at 7 mg and 0.1 g, respectively, the encapsulation efficiency was 90% and the cumulative release rate reached more than 50% within 120 h at pH = 5. This drug delivery system is suitable for loading fat-soluble antineoplastic drugs for sustained release and pH sensitive delivery.

## 1. Introduction

The traditional delivery route for antineoplastic agents is through intravenous administration. This administration leads to low drug concentrations in the lesion area, resulting in poor treatment effect and prolonged treatment cycles [1,2]. The low water solubility and bioavailability of these agents usually requires increased dosage of the drugs in order to achieve the desired therapeutic concentrations in the target area; but high concentrations of drug also affect normal cells, bringing a certain degree of damage to the human body [3,4]. Antitumor drugs delivered by tumor targeting carriers form a targeted drug delivery system, which can concentrate drugs at tumor sites and selectively kill tumor cells [5,6]. This administration method avoids damage to normal cells and has been widely studied.

The enhanced permeability and retention effect (EPR) was first put forward by Meada and Matsumura in 1986 [7]. This study describes a phenomenon by which macromolecules (molecular weight greater than 4 kDa) or nanoparticles (10–800 nm) can pass through the gap of the tumor vessel wall smoothly, as the tumor tissue has good vascular permeability. Moreover, as the tumor lacks lymphatic reflux, macromolecules or nanoparticles entering the tumor cannot be metabolized out, leading to particle accumulation in the tumor [8]. Compared with traditional small molecule chemotherapeutic drugs, macromolecular drugs and nano-drugs remain in the tumor achieving passive targeting. Mesoporous silica is an excellent drug carrier due to its large specific surface area, specific pore volume, and easily modified inner and outer surfaces [9,10]. In addition, the biodegradability of Mesoporous silica nanoparticles (MSN) ensures its safe transport in vivo. Therefore, MSN has been widely used for drug delivery [11,12]. Vallet’s group first applied MSN to drug delivery research in 2001 [13]. The anti-inflammatory drug ibuprofen was loaded into the inner pore of MCM-41 MSN. It was found that the MSN had a high drug loading capacity and showed sustained controlled drug release behavior. Compared with traditional drug carriers, such as polymer nanoparticles and liposomes, it showed higher flexibility, versatility, and stability [14]. These previous studies also indicated that MSN had a high loading rate and sustained release of anti-cancer drugs, such as doxorubicin, paclitaxel, and camptothecin [15,16].

Unlike passive targeting, active targeting is achieved through the interaction of a specific ligand on the drug delivery system with its receptor on tumor cells [17]. Folic acid (FA) is a member of the vitamin B family and is found widely in green leafy vegetables [18]. Studies have found that both free folate and folate conjugates can enter cells through folate receptor-mediated endocytosis [19,20]. Overexpression of the folate receptor has been found on the surface of most malignant tumors, but few exist on the surface of normal cells [21]. FA has the beneficial characteristics of small molecular weight, simple chemical properties, and no immunogenicity, which make it an ideal ligand for targeted cancer therapy [22,23]. FA-modified carrier materials loaded with anticancer drugs can bind to highly-expressed folic acid receptors on the surface of cancer cells, thus increasing the drug concentration at the tumor sites and reducing toxic side effects on normal cells. This makes FA a good candidate ligand in the field of cancer biotherapy, and one of the most popular targeting factors in drug targeting delivery systems [24]. In this study, we prepare an antineoplastic drug carrier by grafting FA to the MSN, endowing the particles with both active and passive targeting characteristics. There is no simple way to graft FA onto MSN directly; therefore, an intermediate that can be grafted with FA and easily connected to the MSN is necessary. Polyethyleneimine (PEI) is a cationic polymer with good cell adhesion [25], though the abundant amino groups on the surface of PEI can cause cell damage. An amidation reaction between the carboxyl groups in FA and the amino groups in PEI minimizes this toxicity while also providing linkage between the molecules.

In this study, curcumin was used as a model drug to evaluate the loading and release performance of MSN-PEI-FA. Curcumin is a chemical constituent extracted from the roots of some plants in the Zingiber and Arisaema families. Curcumin has a variety of pharmacological activities, especially in the prevention and treatment of tumors [26,27]. However, the application of curcumin has been restricted due to poor water solubility and low bioavailability, suggesting that targeted delivery systems could improve its efficacy. In this work, the nano drug carrier MSN-PEI-FA was loaded with curcumin in order to provide a theoretical basis for efficient delivery of liposoluble antineoplastic drugs in the human body.

## 2. Materials and Methods

### 2.1. Materials

Tetraethyl orthosilicate (TEOS), hexadecyltrimethylammonium chloride (CTAC), [3-(2,3-epoxypropoxy) propyl] trimethoxysilane (EPPTMS), poly(ethylene imine) (PEI), folic acid (FA), Curcumin, 1-(3-Dimethylaminopropyl)-3-ethylcarbodiimide hydrochloride (EDC), and *N*-Hydroxysuccinimide (NHS) were purchased from Aladdin Chemical Reagent Co. Ltd. (Shanghai, China). *N*,*N*-dimethylfamide(DMF), NaH_2_PO_4_, Na_2_HPO_4_, ethanol, triethylamine (TEA), and dimethyl sulfoxide (DMSO) were of analytical grade. Human colon cancer cell line (LS174T) was purchased from the Cell Bank of Chinese Academy of Sciences (Shanghai, China).

### 2.2. Preparation

#### 2.2.1. Preparation of MSN

CTAC (2 g) was dissolved in 20 mL of deionized water, then 0.32 mL of TEA was added drop-wise into the above solution, followed by stirring for 2 h at 75 °C. TEOS (1.5 mL) was then added to the mixed solution slowly and the reaction finished after 3 h. The product was washed three times with deionized water and ethanol separately, then dried under vacuum at 60 °C. The product was finally calcined at 600 °C in a muffle furnace for 6 h, and MSN were obtained.

#### 2.2.2. Preparation of MSN-PEI

The method of preparing MSN-PEI was according to the reported work [28]. EPTMS (0.4 g) and PEI (0.5 g) were mixed in 50 mL of DMF solution at 80 °C for 24 h, then 2 g of MSN were added to this solution and allowed to react for another 24 h. The product was washed with DMF and ethanol, then dried in the vacuum freeze dryer.

#### 2.2.3. Preparation of MSN-PEI-FA

To prepare MSN-PEI-FA, the carboxyl groups of FA were activated according to the following steps. FA (0.265 g), EDC (0.24 g), and NHS (0.4 g) were dissolved in 10 mL of DMSO solution and stirred at room temperature for 24 h to activate the carboxyl groups in FA. MSN-PEI (1.0 g) were then added to the mixed solution and stirred for 4 h. The product was washed with ether and freeze-dried.

### 2.3. Characterization of Materials

Scanning electron microscopy (SEM, S4800, Hitachi, Tokyo, Japan) and transmission electron microscopy (TEM, JEM-2100, Lorentz, Tokyo, Japan) were used to record the microstructure of the materials; Fourier-transform infrared spectrometry (FTIR, Tensor II, Bruker, Karlsruhe, Germany). The pore diameters, pore volumes, and surface areas of the carriers were calculated using the BET (Brunauer-Emmett-Teller) method. Zeta potential and particle size were provided by a Zetasizer nano (Malvern Instruments, Worcestershire, UK).

### 2.4. Curcumin Loading Content of MSN-PEI-FA

Curcumin mother solution preparation: 100 mg curcumin was dissolved in 80% ethanol solution and fixed in a 100 mL capacity bottle. A series of curcumin solutions with concentrations ranging from 1 g/mL to 8 g/mL were diluted from the mother solution to establish a standard curve for curcumin responses. The concentration of curcumin was measured with a UV/Vis spectrophotometer at λ = 426 nm. The MSN-PEI-FA (100 mg) were dispersed in 5, 7, and 10 mL of curcumin mother solution. The mixture was stirred overnight at 800 rpm. The solid was obtained by centrifugation and washed with twice with ethanol to remove unloaded curcumin. The encapsulation efficiency (EE) was calculated by Equation (1):

(1) $$\mathrm{EE}\;(\%)=\frac{W_{e}}{W_{0}}\times 100\%$$

where *W_e_* is the amount of drug loaded in MSN-PEI-FA and *W*_0_ is the amount of drug that was first added during the preparation procedure.

The loading efficiency (LE) was calculated by Equation (2):

(2) $$\mathrm{LE}\;(\%)=\frac{W_{e}}{W}\times 100\%$$

where *W* is the weight of MSN-PEI-FA.

### 2.5. Curcumin Release Experiment

In vitro curcumin release from MSN-PEI-FA was studied using three different pH buffer solutions (pH = 5.4, 6.8, and 7.4). PBS solution was composed of Solution A (NaH_2_PO_4_ (0.2 mol/L)) and Solution B (Na_2_HPO_4_ (0.2 mol/L)). To prepare PBS solution of pH 5.4, the pH of Solution A was adjusted to 5.4 with NaOH solution. To prepare PBS solution of pH 6.8, 51 mL of Solution A was mixed with 49 mL of Solution B. To prepare PBS solution of pH 7.4, 19 mL of solution A was mixed with 81 mL of solution B. In the release experiment, 20 mg of curcumin-loaded MSN-PEI-FA were dispersed in 3 mL of the different pH buffer solutions and placed in dialysis bags with a molecular weight cut-off (50 kD), which were sealed and immersed in 47 mL of the correspondent buffer solution. The solutions were transferred to a 37 °C thermostatic oscillator with a shaking rate maintained at 120 rpm. The curcumin content of the buffer solutions was determined by sampling and replenishing the buffer solution at each time point. The concentrations of the curcumin buffer solutions were determined by UV/Vis spectrophotometer at each releasing time point.

## 3. Results and Discussion

### 3.1. Preparation Principle of MSN-PEI-FA

**Figure 1:**
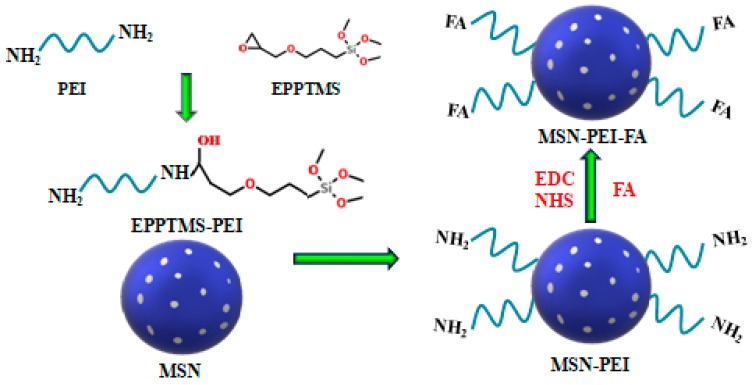
The preparation route and schematic diagram of mesoporous silica nanoparticles- polyethyleneimine-folic acid (MSN-PEI-FA).

The preparation route for MSN-PEI-FA is shown in the schematic diagram of Figure 1. The first step illustrates the reaction between the PEI and EPPTMS. Under alkaline conditions, the epoxy rings in EPPTMS can react with the amino groups of PEI to form EPPTMS-PEI. The silanol groups can then be easily grafted with –OH in MSN. The second step illustrates the reaction between MSN-PEI and FA. There are two carboxyl groups contained in each FA molecule, so FA can graft onto MSN-PEI through an amidation reaction under mild conditions. More concretely, the carboxyl groups in FA were activated by EDC and NHS and it is these activated carboxyl groups that are more likely to form amides with the amino groups.

### 3.2. Characterization

**Figure 2:**
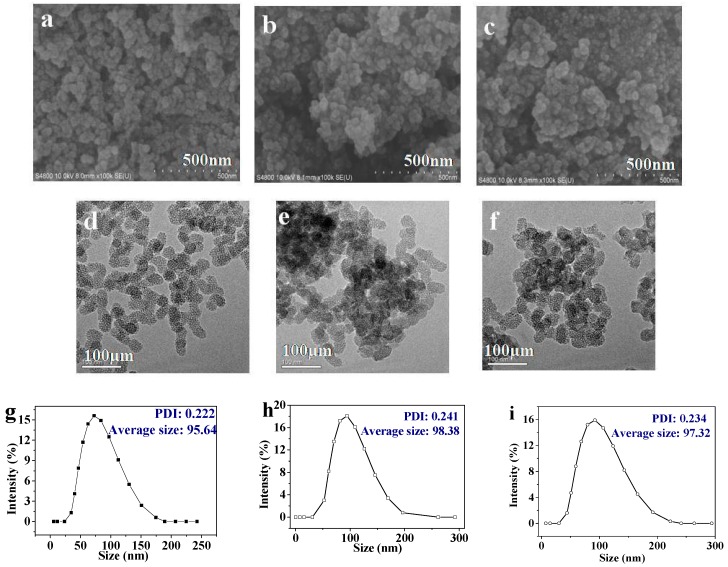
SEM images of (**a**) MSN, (**b**) MSN-PEI, and (**c**) MSN-PEI-FA; the TEM images of (**d**) MSN, (**e**) MSN-PEI, and (**f**) MSN-PEI-FA; the distribution of particle size of (**g**) MSN, (**h**) MSN-PEI, and (**i**) MSN-PEI-FA.

Figure 2a–c show images of MSN, MSN-PEI, and MSN-PEI-FA respectively, from the scanning electron microscope. Because the mesoporous pores of MSN are too small, the parallel arrangement of the pores cannot be observed in the SEM images. The MSN have a high dispersion compared with modified MSN. Figure 2d–f are images of MSN, MSN-PEI, and MSN-PEI-FA respectively, from the transmission electron microscope. It can be seen from the images that MSN are spherical particles arranged in parallel with regular mesoporous structures and diameters of approximately 40 nm. When the MSN were modified with PEI and FA, the particles partially agglomerated, consistent with the results from the SEM. This phenomenon is related to changes in the surface charge of the MSN during modification. The zeta potential of the carriers used in this study was measured to confirm this conclusion. The zeta potential was measured as follows: 5 mg each of MSN, MSN-PEI, and MSN-PEI-FA was dispersed in 500 mL of deionized water. After 30 min of ultrasonication, the zeta potential was determined using Malvern Zetasizer nano. The Zeta potentials of the MSN, MSN-PEI, and MSN-PEI-FA were −55.2 eV, −28.07 eV, and −42.85 eV, respectively, in neutral conditions. PEI contains a large number of amino groups, so the surface charge of PEI is positive. After the MSN were modified by PEI, the absolute value of the Zeta potential of the MSN-PEI is 28.07 eV, lower than that of MSN 55.2 eV. Lower absolute values of the Zeta potentials are not conducive to dispersion of the nanoparticles. When PEI was modified by FA, the absolute values of Zeta potentials of the MSN-PEI-FA increased to 42.85 eV, because the carboxyl groups in FA were now included. Furthermore, the zeta potential of MSN-PEI-FA in PBS (pH = 5.4) was 45.35 eV. When the pH of PBS was 6.8 and 7.4, the zeta potential of MSN-PEI-FA was 42.87 and 40.09 eV, respectively. The particle sizes of the carriers in this work were also measured using a Zetasizer nano. The distributions of the particle sizes are shown in Figure 2g–i. The size of the MSN ranged from 34.77 to 174.73 nm, and the average size was 95.64 nm, maximum content of particles at 72.42 nm was 15.6%, and polydispersity (PDI) was 0.222. After the MSN were modified by PEI, the size of the MSN–PEI ranged from 52.35 to 196.15 nm, and the average size was 98.38 nm, maximum content of particles at 94.15 nm was 18.1%, and PDI was 0.241. The introduction of PEI was the main reason for the increase in particle size, because the introduction of PEI leads to a decrease in the absolute value of the surface charge of the MSN, resulting in aggregation of the nanoparticles. Although the absolute surface potential of the MSN-PEI-FA increased after introducing the FA, the particle size distribution of the MSN-PEI-FA still remained in the range of 44.31–222.67 nm. The maximum content of particles at 92.29 nm was 15.9%, average size was 97.32 nm, and PDI was 0.234.

**Figure 3:**
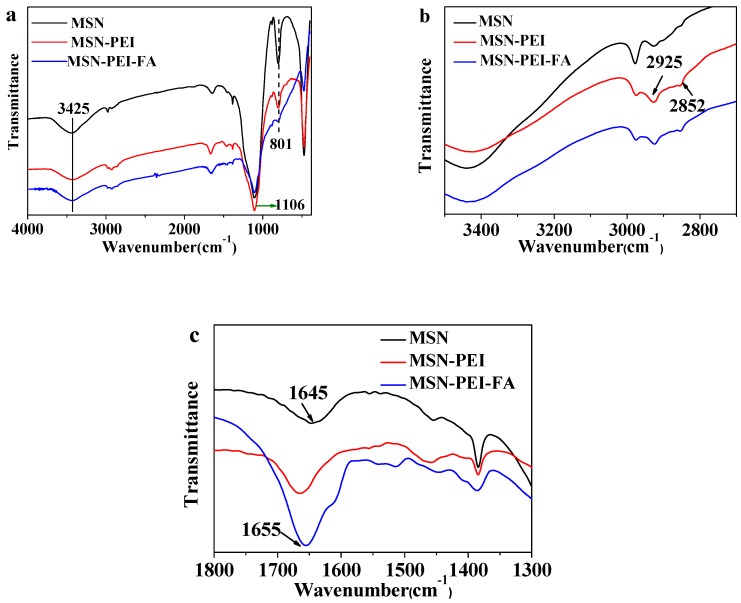
(**a**) The full FTIR spectra of MSN, MSN-PEI, and MSN-PEI-FA; (**b**) the FTIR spectra of MSN, MSN-PEI, and MSN-PEI-FA at 3400 cm^−1^ to 2800 cm^−1^; (**c**) the FTIR spectra of MSN, MSN-PEI, and MSN-PEI-FA at 1800 cm^−1^ to 1300 cm^−1^.

The FTIR spectra are shown in Figure 3a. The strong absorption peak at 1106 cm^−1^ belongs to the stretching vibration of asymmetric Si–O–Si, while the absorption band at 801 cm^−1^ is the stretching vibration of symmetrical Si–O–Si [29,30]. The stretching vibrations of O–H and N–H cause wider absorption peaks at 3425 cm^−1^ [31]. In Figure 3b, the symmetric and asymmetric C–H stretching vibrations correspond to the peaks at 2925 cm^−^^1^ and 2852 cm^−^^1^, respectively [32]. In Figure 3c, the peak at 1645 cm^−^^1^ belongs to the bending vibration of H–O–H [33]. When FA reacts with PEI, a wider absorption band is formed at 1655 cm^−^^1^. This peak belongs to the stretching vibration of C=O in the amide. It also proves that the reaction between FA and PEI is successful.

**Figure 4:**
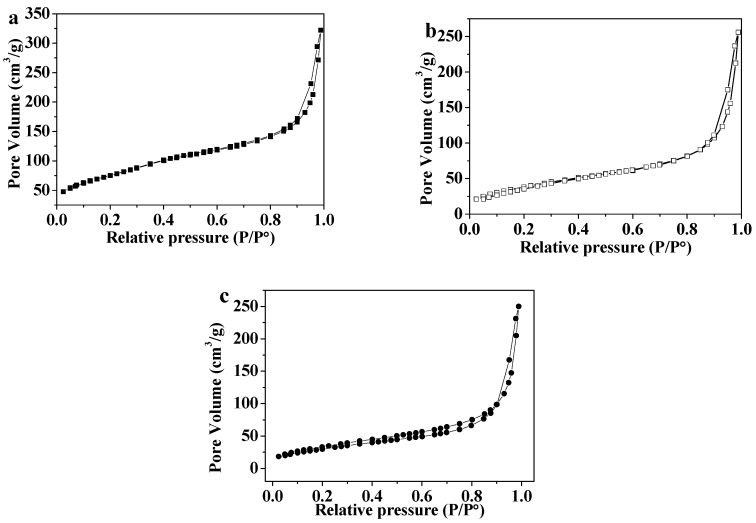
The N_2_ adsorption and desorption curves of (**a**) MSN, (**b**) MSN-PEI, and (**c**) MSN-PEI-FA.

The pore diameters, pore volumes, and surface areas of MSN, MSN-PEI, and MSN-PEI-FA were measured by an N_2_ adsorption and desorption test. The N_2_ adsorption and desorption curves are shown in Figure 4. The N_2_ adsorption and desorption curve types for the three materials are identical and belong to Similar IV-type curves [34]. When P/P_0_ was in the range of 0 to 0.6, N_2_ molecules were adsorbed on the inner surface of the mesoporous material from monolayer to multilayer. Adsorption hysteresis loops occurred when P/P_0_ increased from 0.8 to 0.95, which corresponded to the capillary condensation system of porous adsorbents. When P/P_0_ was in the range of 0.95 to 1.0, the surface multilayer adsorption occurred. The surface areas of MSN, MSN-PEI, and MSN-PEI-FA were calculated by the multipoint BET (Brunauer-Emmett-Teller) method [35], and the results are shown in Table 1. The average pore diameters and the total pore volumes of MSN, MSN-PEI, and MSN-PEI-FA were calculated by the BJH (Barrett, Joyner, and Halenda) method [36]. The results indicate that after the MSN were modified with PEI and FA, the surface areas, pore diameters, and pore volumes all declined. This is because the PEI and FA occupy pores of the MSN in the modification process.

### 3.3. Optimizing the Encapsulation Efficiency and Loading Efficiency

The loading and encapsulation efficiencies are important factors in evaluating a drug delivery system and the results for MSN and MSN-PEI-FA with different curcumin dosages measured in this work are shown in Table 2. With increasing doses of curcumin, the loading efficiency by MSN and MSN-PEI-FA increased because curcumin is more likely to bind to loading sites when the concentration is high. However, the increasing concentrations of curcumin used in loading the MSN -PEI-FA also caused the unloaded of curcumin to increase. When the curcumin dosage increased from 5 mg to 10 mg, the loading efficiency of MSN-PEI-FA increased from 4.98% to 7.96%, while the encapsulation efficiency decreased from 99.79% to 79.57%. Considering the loading efficiency and encapsulation efficiency, we selected an experimental dosage of 7 mg curcumin for this work. The loading efficiency and encapsulation efficiency of the MSN for curcumin is significantly higher than that of the MSN-PEI-FA. This is because FA and PEI occupy some of the pores on the MSN, reducing the loading efficiency of the curcumin.

### 3.4. The Release Rate at Different pH Values

**Figure 5:**
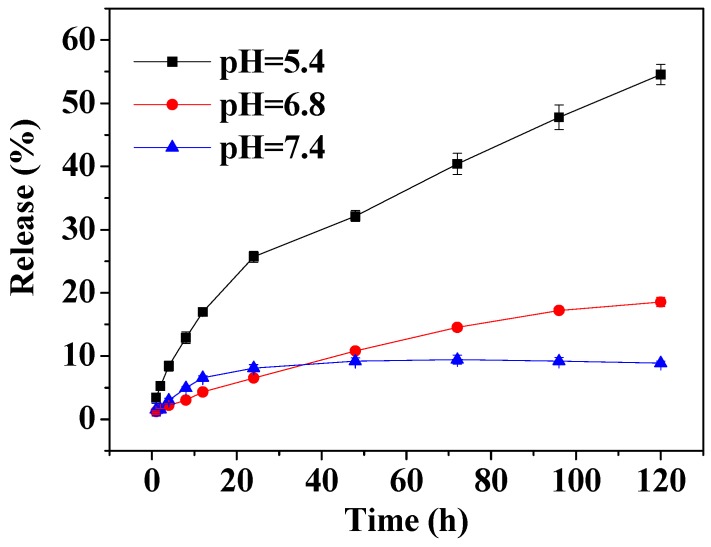
The release rate of curcumin loaded in MSN-PEI-FA at different pH values.

The pH value of normal tissue in the human body is 7.4, while that of tumor tissue is approximately 6.8, and that of tumor cells is lower still (5.0–5.5). This is due to the large amount of lactic acid produced through anaerobic respiration of the tumor cells. In this work, the in vitro drug release experiments were carried out under three different conditions. Specifically, MSN-PEI-FA/curcumin was investigated in PBS buffer at pH 7.4, 6.8, and 5.4. The release rate and release time curves are shown in Figure 5. The total release rates at pH 7.4, 6.8, and 5.4 within 120 h were 8.87%, 18.52%, and 54.56%, respectively. The release rate of curcumin in normal tissues was very low. The release rate of curcumin gradually increased with decreasing pH and the maximum release rate reached greater than 50%. Therefore, MSN-PEI-FA display sustained release and pH-sensitivity, achieving the release of curcumin in the tumor microenvironment.

### 3.5. Cytotoxicityand Uptake by Tumor Cells

**Figure 6:**
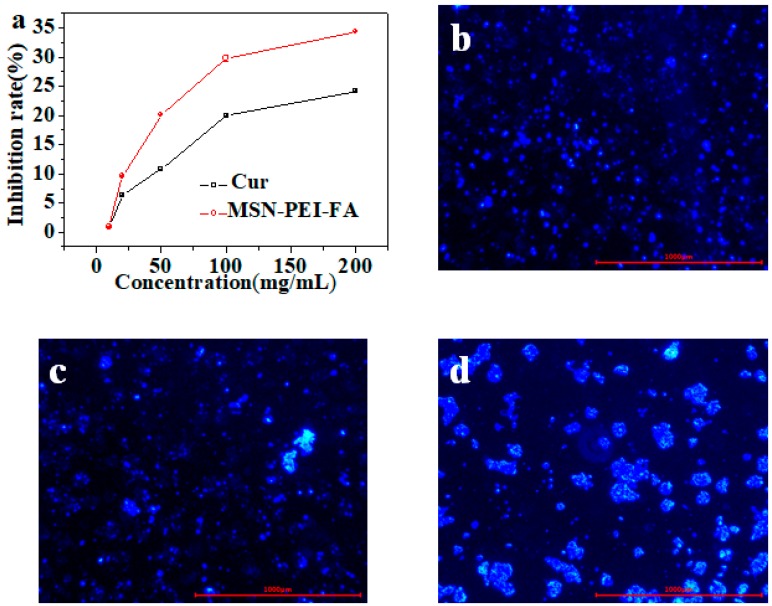
(**a**) Inhibition rate of Cur and MSN-PEI-FA/Cur; fluorescence microscopic images of LS174T for coumarin-loaded MSN (**b**), MSN–PEI (**c**), and MSN–PEI-FA (**d**) intake experiments.

The MTT method was used to evaluate the cytotoxicity of MSN-PEI-FA/Cur on colon cancer cells. MSN-PEI-FA/Cur solution of concentrations 10–20 µg/mL was prepared in PBS. The rate of inhibition (%) of colon cancer cells by MSN-PEI-FA/Cur was calculated using Equation (3). The results are shown in Figure 6a. A high concentration of MSN-PEI-FA/Cur exerted high cytotoxicity on colon cancer cells. Furthermore, the toxicity of MSN-PEI-FA/Cur was higher than that of Cur at each concentration. This is due to the presence of FA, because of which the uptake of MSN-PEI-FA/Cur nanoparticles by tumor cells was significantly enhanced, resulting in increased cytotoxicity.

(3) $$\mathrm{Inhibition}\left(\%\right)=100\%-\left(\frac{{\mathrm{OD}}_{\mathrm{drug}}}{{\mathrm{OD}}_{\mathrm{contrast}}}*100\%\right)$$

To evaluate cell uptake of particles, coumarin 6, which has a structure similar to that of curcumin, was used as a fluorescent dye and embedded in MSN-PEI-FA to study the uptake of vectors by colon cancer cells. The method of loading coumarin on carrier was similar to that of curcumin. Different carriers of MSN, MSN-PEI, and MSN-PEI-FA (50 mg) were dispersed in 10 mL of coumarin solution at a concentration of 50 µg/mL, respectively. The mixture was stirred overnight at 800 rpm. The pellet obtained by centrifuging the sample was washed with CH_2_Cl_2_ and ethanol to remove unloaded coumarin. Carriers containing coumarin were incubated in a 6-well plate containing colon cancer cells; the results were recorded using a fluorescence inversion microscope. As shown in Figure 6b–d, the uptake ability of MSN-PEI-FA by tumor cells was significantly higher than that of MSN and MSN-PEI. It confirms that the introduction of FA into carriers can increase the uptake rate of cancer cells.

## 5. Conclusions

This work demonstrates the successful modification of MSN through the grafting of PEI and FA. The loading of curcumin to FA-modified MSN as the drug carrier was further studied. The drug release behavior of MSN-PEI-FA was also studied as a function of pH. The loading and encapsulation efficiencies of MSN-PEI-FA for curcumin are 6.46% and 92.13%, respectively, which are lower than those obtained with MSN. This is because the FA and PEI occupy some of the pores of the MSN, affecting the loading efficiency of the porous carriers. Drug release experiments show that MSN-PEI-FA has an obvious sustained release behavior and the cumulative release rate under a microacidic environment is significantly higher than that under neutral conditions. FA-modified MSN were successfully prepared and in this study, their advantages and prospects for fat-soluble anticancer drug delivery are highlighted.

## Figures and Tables

**Table 1:** The corresponding parameters of N_2_ adsorption and desorption.

Material	Pore Size (nm)	Pore Volume (cm^3^)	Surface Area (m^2^/g)
MSN	2.58	2.52	274.3
MSN-PEI	2.19	0.42	145.03
MSN-PEI-FA	2.02	0.40	140.15

**Table 2:** The effect of different curcumin dosages on encapsulation efficiency and loading efficiency.

Carrier	Dosage (g)	Curcumin (mg)	EE%	LE%
MSN	0.1	5	99.86 ± 0.24	4.99 ± 0.01
	0.1	7	93.33 ± 1.23	6.60 ± 0.09
	0.1	10	81.4 ± 0.7	8.14 ± 0.07
MSN-PEI-FA	0.1	5	99.79 ± 0.31	4.98 ± 0.02
	0.1	7	92.13 ± 1.27	6.46 ± 0.09
	0.1	10	79.57 ± 1.49	7.96 ± 0.15
