# Erratum: Sun, X.; et al. Folic Acid and PEI Modified Mesoporous Silica for Targeted Delivery of Curcumin. *Pharmaceutics*, 2019, *11*, 430

**DOI:** 10.3390/pharmaceutics12070623

**Published:** 2020-07-03

**Authors:** Xiaoxiao Sun, Nan Wang, Li-Ye Yang, Xiao-Kun Ouyang, Fangfang Huang

**Affiliations:** School of Food and Pharmacy, Zhejiang Ocean University, Zhoushan 316022, China; idsxx799@163.com (X.S.); ynwangnan@163.com (N.W.); liyey@zjou.edu.cn (L.-Y.Y.); gracegang@126.com (F.H.)

The authors wish to make the following corrections to this paper [1]:

1. In Figure 2d–f, the TEM images of (d) MSN, (e) MSN-PEI, and (f) MSN-PEI-FA ruler had an error in the unit when enlarging the annotation;
2. In Figure 6a, there is something wrong with the mark of concentration unit of Cur;
3. The dose of Cur in the experiment was from 10 to 200 µg/mL, but it was mistakenly written as 10–20 µg/mL in the manuscript.

After the publication of this work, we noted the mistake and issued an erratum for correction. The corresponding sentence, Figures 2 and 6a have now been corrected in this erratum.

“The MTT method was used to evaluate the cytotoxicity of MSN-PEI-FA/Cur on colon cancer cells. MSN-PEI-FA/Cur solution of concentrations of 10–200 µg/mL was prepared in PBS”.

The authors would like to apologize for any inconvenience caused to the readers by these changes.

## Figures and Tables

**Figure 2:**
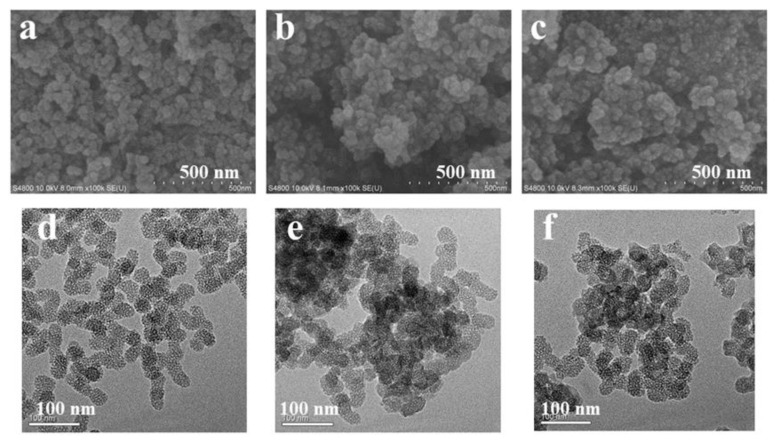
SEM images of (**a**) MSN, (**b**) MSN-PEI, and (**c**) MSN-PEI-FA; the TEM images of (**d**) MSN, (**e**) MSN-PEI, and (**f**) MSN-PEI-FA.

**Figure 6:**
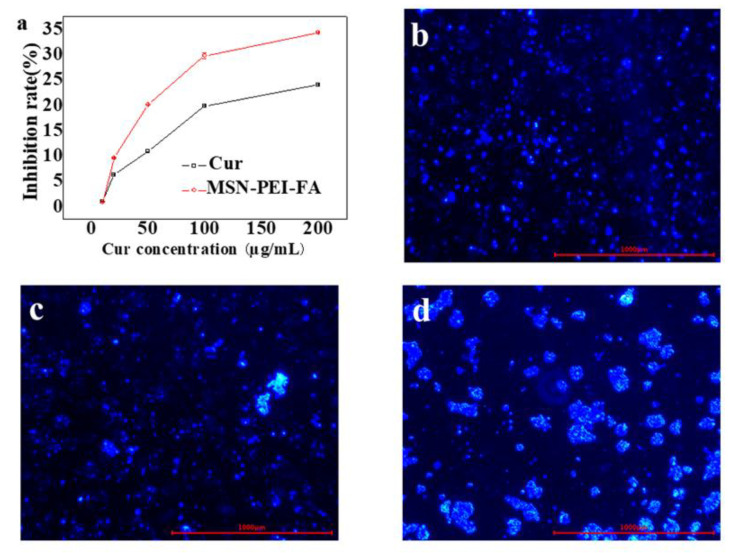
(**a**) Inhibition rate of Cur and MSN-PEI-FA/Cur; fluorescence microscopic images of LS174T for coumarin-loaded MSN (**b**), MSN–PEI (**c**), and MSN–PEI-FA (**d**) intake experiments.
